# Nurse-Driven Interventions Reduce Central Line-Associated Bloodstream Infection Close to Zero in One Pediatric Oncologic Facility: A Single-Center Retrospective Observational Study

**DOI:** 10.3390/nursrep14040197

**Published:** 2024-09-26

**Authors:** Federico Turoldo, Antonella Longo, Mariavittoria Sala, Denis Valentini, Nicole De Vita, Sara Toniutti, Loredana Zuppel, Natalia Maximova

**Affiliations:** 1 Department of Medicine, Surgery and Health Sciences, Hygiene and Preventive Medicine, University of Trieste, Strada di Fiume 447, 34149 Trieste, Italy; federico.turoldo@studenti.units.it; 2 Department of Pediatrics, Institute for Maternal and Child Health—IRCCS “Burlo Garofolo”, Via dell’Istria 65/1, 34137 Trieste, Italy; antonella.longo@burlo.trieste.it (A.L.); nicole.devita@burlo.trieste.it (N.D.V.); sara.toniutti@burlo.trieste.it (S.T.); loredana.zuppel@burlo.trieste.it (L.Z.); 3 Department of Public Health and Pediatric Sciences, University of Turin, 10136 Turin, Italy; mariavittoria.sala@unito.it; 4 Department of Emergency Medicine, Hospital of Cattinara, Azienda Sanitaria Universitaria Giuliano Isontina (ASU GI), Strada di Fiume 447, 34149 Trieste, Italy; denis.valentini@asugi.sanita.fvg.it

**Keywords:** central venous catheter, pediatric, oncological, central line-associated bloodstream infections, catheter-related bloodstream infections

## Abstract

Background: Central line-associated bloodstream infections (CLABSIs) are critical infectious complications among pediatric hematology-oncology patients, and the management of central venous catheters (CVCs) by healthcare personnel can significantly influence the incidence of these infections. This study evaluates the impact of nurse-led changes in CVC management on the incidence of CLABSIs. Methods: This single-center, retrospective observational study was conducted at an urban, tertiary referral, and academic center serving pediatric patients. Results: The study cohort comprised 239 patients and 323 CVCs seen between 2012 and 2022. CLABSI was defined according to the Centers for Disease Control and Prevention definitions. Oncology nurse leaders developed CVC-specific educational modules for CLABSI prevention. All the relevant information during the CVC maintenance period was noted in the patient’s CVC logbook. A total of 24 (7%) cases of confirmed CLABSI were identified. The incidence of CVC-related infections was 0.32 cases per 1000 catheter days (95%CI: 0.19–0.45). The incidence decreased by 40% between the first and second three-year study period. Documented exit-site infection was reported in 32 (10%) cases. The correlation between exit-site infection and CLABSI was found in 9 (28%) cases. Our CVC-related infection rates are significantly lower than the incidence reported by the Italian Association of Pediatric Hematology and Oncology, which settles at 3–5 cases per 1000 catheter days. Conclusions: Our data confirm the effectiveness of local CVC management guidelines in preventing CVC-related infectious complications.

## 1. Introduction

Central line-associated bloodstream infections (CLABSIs) and catheter-related bloodstream infections (CRBSIs) are the most critical central line-correlated infectious complications among pediatric hematology oncology patients [1,2]. The incidence of CLABSI among pediatric oncological patients is 1–4.6 per 1000 catheter days for external central venous catheter (CVC) engaging about 25% of this population, with an estimated mortality rate between 12.5% and 25% [3,4,5]. Patients are particularly susceptible to infectious complications during episodes of chemotherapy-induced neutropenia, but also non-neutropenic outpatients can suffer from CVC-related infections [6,7].

The incidence of CLABSI is variable according to modifiable and non-modifiable factors. If patient characteristics such as age, low body weight, disease status, and comorbidities cannot be changed, the “human factor”, which determines how a CVC is managed, is improvable. The correct management of the central line, the proper preparation and administration of infusion liquids, the correct dressing techniques, and appropriate drug delivery methods are all modifiable factors that have a certain weight in the incidence of catheter-related infections [8,9,10]. In our Institute, CVC management is the responsibility of dedicated nursing staff, per standard practice. The decision to conduct this study was dictated by the need to verify whether we can reduce CVC-related infectious complications thanks to the rigorous and collaborative application of local CVC management guidelines.

After introducing the CVC management protocol, designed by highly qualified oncology nurse staff, and its application in all satellite hospitals, we aimed to estimate the incidence of CVC-related infections, placing this question as the study’s primary aim.

## 2. Materials and Methods

### 2.1. Study Design and Population

This is a single-center retrospective observational study. The study reviewed CVC data routinely collected from January 2012 to July 2022. Three hundred twenty-six consecutive children and adolescents with cancer and hematological disease who initiated care at the Department of Pediatric Hematology Oncology and Bone Marrow Transplantation of the urban, tertiary referral academic medical center Burlo Garofolo Children’s Hospital were included in the study group. Inclusion criteria were as follows: CVC inserted in our hospital between January 2012 and July 2022, hematological or oncological diagnosis, age less than 18 at the time of CVC insertion, and informed consent for using clinical data signed by parents at admission. Exclusion criteria were CVC removal within the first 24 h of insertion and personal CVC logbook unavailability.

This was a unit-based quality improvement project designed by nurses with long-standing experience. The study’s primary aim was to assess whether the nurses’ CVC management recommendations would result in a lower incidence of infectious complications than the rates reported in the literature. The secondary aim was to determine if, among the analyzed variables, it was possible to identify any additional risk factors for CVC-related infectious in a pediatric oncological setting.

Demographic and clinical characteristics data were collected on the patients with an oncologic or hematologic diagnosis who had a CVC placed.

### 2.2. Ethical Approval and Informed Consent

The study protocol was approved by the Institutional Review Board of the IRCCS Burlo Garofolo (reference No. IRB RC 32/2023), and the study was conducted following the Declaration of Helsinki. The data were collected according to the Authorization to Process Personal Data for Scientific Research Purposes (Authorization No. 9/2014) [11].

According to our Institute regulations, parents or guardians had to give written informed consent on the first visit to use any clinical data in research. The medical records of all the patients were analyzed individually and anonymously.

### 2.3. Nurse-Led Quality Improvement CVC-Management Protocol

**Figure 1:**
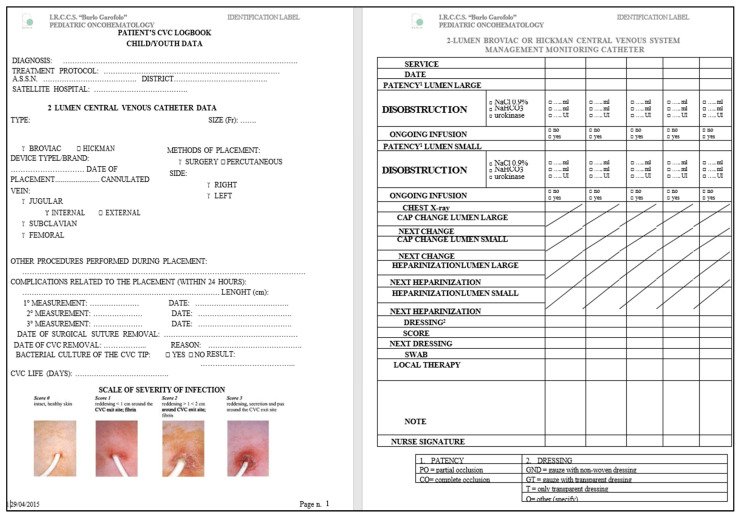
The personal logbook is given to the patient or parent and used by the nursing personnel to add information about placement, dressing, eventual dressing preferences, maintenance problems, and any infectious event reported.

Following a critical evaluation of the best evidence, the oncological nurse team developed changes to the unit’s nursing practice on CVC handling guidelines to reduce CLABSI incidence. The differences in the modified guidelines compared to the in-force departmental CVC maintenance protocol were the following:

- The ordinary management of the CVC is exclusively the responsibility of the trained personnel;
- Two-person dressing approach;
- Use an aseptic technique during any CVC manipulation that requires the lumen opening or removal of the CVC exit-site dressing;
- The preparation of injecting drugs or solutions to be infused into the CVC under a laminar flow hood using an aseptic technique;
- A total of 2% chlorhexidine and 70% isopropyl alcohol disinfection of needle-free connectors (NFCs) and cover with a double layer of sterile gauze for biological fluid contamination protection;
- The maintenance of the closed circuit during the use of infusion lines;
- The replacement of infusion lines every 24 h if blood products, total parenteral nutrition (TPN), propofol administration, or closed-circuit interruption; otherwise, replacement is carried out every 72 h;
- The production of the patient CVC logbook, which includes placement information, dressing according to the eventual patient’s preferences, any maintenance problems, and any infectious event reported (Figure 1);
- Newly hired and satellite hospital personnel training to prepare them to work independently, maintaining uniform CVC management.

### 2.4. Central Line Data

We documented all the CVCs, along with the device type, number of lumens, tube size, date of placement, laterality, cannulated vein, duration of CVC, dressing frequency, CVC use (TPN, transfusions, blood sampling, or apheresis), and removal indication. CVCs that had been in place for less than one day were excluded. If a patient underwent multiple CVC placements, it was considered a different entry in the database. CVCs were categorized as tunneled (cuffed and non-cuffed), non-tunneled, and implanted subcutaneously.

### 2.5. CVC Dressing Protocol

CVC dressings were performed according to the GAVeCeLT protocol (Italian expert group on long-term central venous accesses). They were changed every seven days (planned) or whenever they became damp, detached, or visibly soiled (unplanned). If the dressing and CVC exit site appeared perfect in inpatients, the dressing was postponed to 10 days.

After the adhesive tissue was removed with Remove© wipes (Smith and Nephew, Watford, England, UK ), the dressing area’s skin was cleaned using a BD ChloraPrepTM applicator (Becton, Dickinson and Company, Franklin Lakes, NJ, USA) that delivers a solution of 2% *w*/*v* chlorhexidine gluconate and 70% *v*/*v* isopropyl alcohol without orange dye. BD Chloraprep™ was applied by rubbing the skin around the exit site back and forth to cover the whole dressing area and let dry for 30 s. When the skin was dry, adhesive transparent dressing IV3000 (Smith&Nephew, Watford, UK) or Tegaderm™ I.V. Advanced (3M, Maplewood, MN, USA) was attached.

### 2.6. CVC-Associated Infectious Data

CLABSI is defined as a culture of recognized pathogens from one or more blood cultures, with the organism not related to an infection at another site, in patients with a CVC in place within 48 h before detection. If a common skin contaminant is cultured, two or more blood cultures drawn on separate occasions are required, along with specific symptoms [12]. We considered CLABSI in the inpatient setting if the first positive blood culture occurred >48 h after admission or <48 h after hospital discharge. In the outpatients, CLABSIs were considered if the first positive blood culture occurred >48 h after hospital discharge or <48 h after admission [13]. We considered the mucosal barrier injury to reduce the potential overestimation of true CRBSI incidence [14]. The occurrence of skin irritation was defined as the presence of areas of skin loss, erythematous plaques, or vesicles at the site of CVC insertion. We have defined a CVC exit-site infection as hyperemia, induration, and/or tenderness ≤ 2 cm from the catheter exit site with negative blood cultures. The exit site was evaluated using a visual exit-site score [15].

### 2.7. Statistical Analysis

Data were statistically analyzed using the R software version 4.3.1 (16 June 2023 ucrt) for Windows [16].

Descriptive statistics were used to analyze the demographic data, CVC characteristics, and details on CVC insertion, usage, and complications using the “gtsummary” package [17]. Categorical data were presented as frequency and percentage, while continuous data were presented as the median and interquartile range (IQR). The tests defaulted were the Wilcoxon rank sum test for continuous variables, Pearson’s Chi-squared test without Yates’ correction for categorical variables with all the expected cell counts ≥ 5, and Fisher’s exact test for categorical variables with any expected cell count < 5. Incomplete observations were removed from the analysis.

The incidence of infection was calculated as the number of cases/person-time, and the 95% CI was calculated using the formula CI = rate ± 1.96 × sqrt (rate/person-time).

In order to study additional factors associated with infection, the dataset was divided according to CLABSI, and also alternative outcomes were proposed and explored to conduct sensitivity analyses. In this secondary analysis, associations were explored by running a univariate logistic regression on the relative outcome for each variable taken into consideration, and the resulting OR and 95%CI were reported. In the supplements, a survival analysis was conducted to establish the cumulative incidence of infection among different groups of interest with the help of the “survival” package [18].

The statistical significance was arbitrarily set to <0.05.

## 3. Results

### 3.1. Patient Population and Device Characteristics

In total, 239 pediatric oncological and hematological patients had a CVC inserted between January 2012 and December 2019, with the last follow-up occurring in December 2022. Some patients had multiple CVC placements, often at different times, with potential variations in patient characteristics or the CVC itself. For this reason, each CVC placement was considered a separate entry. The total number of CVCs inserted during the study period was 323, with 66 (20%) patients requiring one or more CVC replacements. The most common primary diagnoses were acute lymphoblastic leukemia (*n* = 145, 45%) and solid tumors (*n* = 100, 31%). In total, 122 (37.8%) patients underwent hematopoietic stem cell transplantation (HSCT), and 245 (75.9%) patients received TPN.

All the CVCs were inserted according to the local standard operating procedures. The median age at placement was 6.7 years (range, 0–18). The most common central device type (*n* = 210, 6%) was Broviac^®^—Hickman^®^ (Bard Access Systems). All the central devices were tunneled and cuffed. The median lumen density was 1.46, and the median duration of the central device was 228 days (IQR, 103–320). The following indications for removal were observed: 200 (62%) due to end of therapy, 31 (10%) due to CVC-related infections, 31 (10%) due to malposition and dislodgement, 27 (8.5%) due to death, 19 (6%) due to occlusion and malfunction, and 11 (3.5%) due to breakage. Patient and central device characteristics are shown in Table 1.

### 3.2. Central Line-Associated Infectious Events

During the study period, we identified 24 proven CLABSI events in the hospital’s electronic database. The rate of CLABSI was 0.32 infections per 1000 catheter days (95%CI: 0.19–0.45). We analyzed patient and CVC characteristics to find the factors that might increase the risk of infection (Table 2).

**Figure 2:**
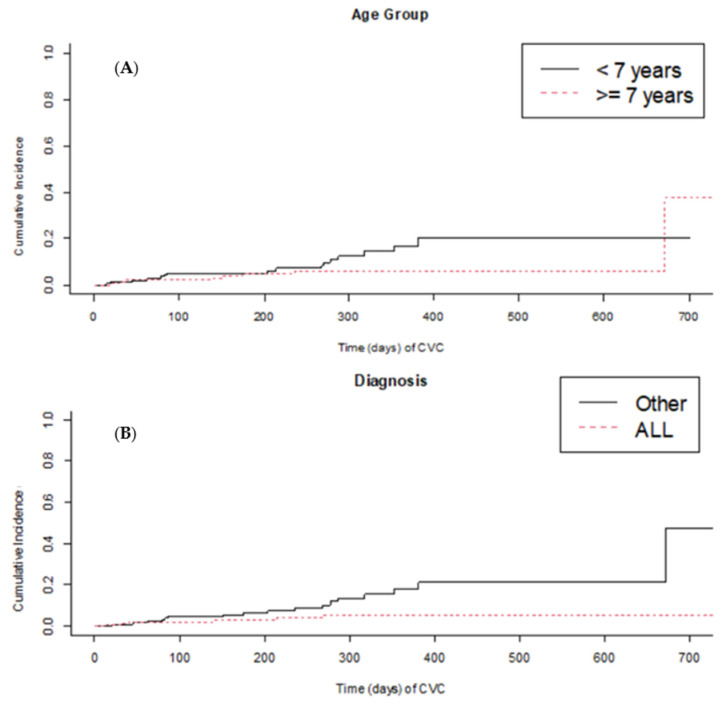
CVC-related infections and patients’ variables. (**A**) Age ≥ seven years appears to be a protective factor for CLABSI without reaching statistical significance (*p* > 0.05). (**B**) ALL diagnosis was associated with a lower incidence of CLABSI (*p* = 0.042).

Only ALL diagnosis was associated with a lower incidence of CLABSI (*p* = 0.042). Even if no other statistically significant association with infection was found, older age (>seven years) and insertion in the subclavian vein appear to be protective factors (Figure 1). We found no correlation even between CLABSI and total parenteral nutrition (TPN) or CVC occlusion events in our cohort. The reliability of the results was tested through sensitivity analysis. The sensitivity analysis was also extended to patients with possible CVC-related infection and CVCs removed due to infection (Table 3). Fifty-one cases of possible infection were identified, and in this population, the patients’ ages were also lower than the mean cohort age. The incidence of possible infection was 0.67 events per 1000 days of CVC (95%CI: 0.49–0.86). Thirty-one infected CVCs (10%) were removed because of the infection. No other significant association between infection and patient or CVC characteristics was found. Figure 2 shows a survival analysis to investigate the cumulative incidence of CLABSI among the patients, focusing on age and ALL diagnoses.

The majority of the germs involved in the CLABSI were Staphylococcus aureus (45%), Staphylococcus epidermidis (17%), Pseudomonas aeruginosa (13%), Candida albicans (13%), and Candida parapsilosis (13%). Ten percent (32 events) of all the CVCs analyzed had a positive exit-site culture, with the prevalence of Staphylococcus aureus (50%). The skin site colonization and CLABSI were correlated in nine cases (28%). A strong correlation was found between CVC tip colonization and bloodstream infection: the same pathogen was isolated from blood cultures in 11 out of 18 cases (61%).

### 3.3. Dressing and CVC Exit-Site Score

We assessed whether the frequency of dressings correlates with CVC-related infections. The median percentage of dressings with 1–6 day intervals was significantly higher in the CLABSI group than in the non-infected group (50% of total dressing, IQR 37–68% vs. 30%, IQR 22–43%; *p* < 0.001). The median percentage of dressings performed once a week was significantly higher in the non-infected group compared to the CLABSI group (50% of the total dressing, IQR 38–61% vs. 31%, IQR 11–45%; *p* < 0.001). No significant differences were found in both groups for dressing performed every ten days (16% of the total dressing, IQR 9–24% vs. 12%, IQR 5–28%, *p* = 0.3). We obtained comparable, statistically significant results by analyzing the correlation between the frequency of dressings and the number of CVCs removed due to infection: 54% vs. 29% for 1–6 day intervals and 31% vs. 50% for once weekly dressing in the CLABSI group and the non-infected group, respectively; *p* < 0.001. To further explore the relationship between dressing frequency and infection, we calculated different infection rates in the subsets of patients whose percentage of dressings falling within the 8–10 days range exceeded a variable threshold. We defined this subgroup of patients with more than 20% of dressing in the 8–10 days range as “less frequent dressing”. Table 3 illustrates the infection rate in both groups; there was no statistically significant difference (two-sample test for Poisson rates *p*-value = 0.075). In the supplements, we tested different threshold values. The infection rate initially reduced even if the patients were subject to less frequent dressing, and then seemed to stabilize around the value of 0.495 cases per 1000 CVC days considering patients who were dressed with an 8–10 days range of more than 30% of the time (Figure S1).

In the event of a suspected or confirmed exit-site infection, it is customary to perform CVC dressings in the pediatric hematology oncology department. Patients with a CVC exit-site score of 0 and without malfunction problems received the medication at home from the appropriately trained staff of the satellite hospitals. For this reason, the relationship between the dressing location and CLABSI was predictable. The median percentage of dressings performed at our department on patients with CLABSI compared to other locations was significantly higher (100%, IQR 69–100% vs. 0%, IQR 0–31%; *p* < 0.001).

We investigated a possible relationship between the visual exit-site score grading and CLABSI. A statistically significant correlation exists between CLABSI and exit-site score ≥ 1. In the patients without CLABSI, the median number of dressings with a score of 0 was 0.93 compared to 0.81 in the CLABSI group (*p* = 0.001).

### 3.4. CVC Removal Infection-Related Indication

Thirty-one (9.6%) CVCs were removed due to CLABSI. The only variables correlating with CVC removal for infection are ALL and long-time catheterization. ALL diagnosis is a protective factor, with only 26% of the CVCs removed due to infection compared to 47% removed due to other reasons (*p* = 0.025). CVCs that remain in placement longer are frequently removed for reasons other than infection (*p* = 0.019). Other variables, such as CVC model and type, method of insertion, number of lumens, HSCT, TPN, and occlusion events, are not correlated with infection-related removal indication (Table S1).

## 4. Discussion

The literature data report an incidence of central CLABSI between 1.7 and 11.3 cases for 1000 days catheter. In the oncology and hematology wards, the incidence is 1–4.6 per 1000 catheter days for external CVCs. CLABSI affects about 25% of pediatric patients with oncological and hematological disease, and its estimated mortality rate is between 12.5% and 25% [3]. Infected CVC management differs in pediatric patients compared to adults, particularly in the oncology setting. The necessity of peripheral vein cultures for diagnosing CVC-related infection remains controversial in children because of the poorer venous assets. Conservative, pharmacologically focused management through CVC remains mandatory, with CVC removal only in selected cases [19]. Therefore, the prevention of CLABSI in this setting of patients becomes a more effective approach.

The nursing initiative was carried out to reduce the incidence of CLABSI through education and bundled intervention implementation in our pediatric oncology hematology department. Their effort and determination achieved significant and sustained decreases in CLABSI over ten years, bringing the incidence consistent with national data to 0.32 proven events for 1000 catheter days only. The protocol’s capillary distribution within the satellite hospitals that manage the patients treated in our department and the frequent retraining of the healthcare teams were probably among the most important factors contributing to its effectiveness.

The creation of the CVC logbook is an innovation that allows healthcare workers in any hospital to safely manage every central device inserted in our department. This logbook is compiled for each CVC placed in our department. It reports all the insertion details and specifics of each dressing performed in and outside the department beyond occlusion, dislocation, or exit-site infection episodes. Our study is the first report in the literature of a personal CVC logbook filled out at each notable catheter handling. Another critical point is carefully evaluating the exit site during each CVC dressing. A section where the exit site is classified according to the Visual Exit Site Score has been inserted in the CVC logbook [20]. This tool helps identify the early signs of local infection and prevents microorganism migration and the colonization of the CVC, avoiding CLABSI.

In our cohort, 32 (10%) of the 323 catheters with a positive exit-site swab resulted in a match with a positive blood culture. Of these, only nine (28%) presented with CLABSI, which means that less than one-third of exit-site infections resulted in an extraluminal migration of the pathogen. The sterile technique of handling the infusion lines under a laminar flow hood guarantees a low risk of intraluminal bacterial dissemination.

TPN, multi-lumen devices, chemotherapy treatment, immunosuppression, and the number of days of catheterization were recognized as modifiable risk factors that increased the probability of developing CLABSI [21]. Among the non-modifiable factors is known significant negative surviving correlation with diabetes mellitus, cardiovascular disease, lung disease, chronic kidney disease, the presence of ≥3 comorbidities, gut or skin graft versus host disease, patients within 15 days after stem cell transplant, high-risk neuroblastoma, AML, relapsed ALL, and dressing/line concerns or issues within the past 72 h [22,23]. In our study, only a few variables were associated with infection. Presumably, such a low incidence of infections affected the identification of risk factors and discouraged the implementation of further analysis. The association between ALL and a lower infection rate is probably linked to the intensity of chemotherapy protocols. While other malignancies have a strictly programmed time interval for chemotherapy cycles, ALL protocols require a normalized white blood cell count before starting a new cycle, thus exposing patients to a milder infectious risk. In the years ahead, by expanding the data pool, it could be possible to investigate the risk factors and patterns of infection in our setting to be included in new surveillance protocols. Furthermore, even if some patients underwent less frequent dressing, they were adequately selected since the incidence of infection did not change considerably. In fact, in patients who underwent more than 20% of dressing in a range of 8–10 days, proven infections were 0.314 per 1000 days of CVC, similar to the incidence in the whole cohort.

Although we comprehensively examined the factors that led to the significant reduction in CLABSI, our study has several limitations. First, it is a retrospective, one-facility study. Given our cohort’s low incidence of CLABSI, a much larger sample size is needed to perform multivariate analyses, considering all the variables examined. Future projects could explore some interesting data on the life of the catheter that have yet to be considered, such as the influence of different therapeutic protocols.

## 5. Conclusions

In conclusion, given our department’s meager infection rate, this study provides important insights: it highlights the effectiveness of our nurse-driven protocol and demonstrates how meticulous attention and the introduction of new tools, such as a personal CVC logbook, contribute to positive patient outcomes. Targeted interventions with protocols produced by highly trained professionals, their sharing, and the intensive training of satellite hospital staff led to a significant drop in infectious complications among pediatric hematological oncology patients with CVC. This example will provide readers with a practical tool for implementing similar protocols in their settings.

## Figures and Tables

**Table 1:** Main characteristics of the population.

Characteristic	Frequency ^1^ (*n* = 323)
Sex	
- Female	121 (37.5%)
- Male	202 (62.5%)
Age (years)	6.71 (2.95–11.90)
Duration of CVC (days)	228.0 (103.0–320.5)
Total CVC placements ^2^	
- 1 CVC placement	173 (72.4%)
- 2 CVC placements	50 (20.9%)
- 3 CVC placements	14 (5.9%)
- 4 CVC placements	2 (0.8%)
Lumen	
- Monolumen	175 (54.2%)
- Bilumen	148 (45.8%)
Diameter of CVC (French)	8.00 (6.00–9.00)
Diagnosis	
- Acute lymphatic leukemia	145 (44.9%)
- Acute myeloid leukemia	22 (6.8%)
- Myelodysplastic syndromes	26 (8.0%)
- Non-malignant hematological diseases	26 (8.0%)
- Solid Tumors	100 (31.0%)
- Unknown	4
HSCT	
- Yes	122 (37.8%)
- No	201 (62.2%)
TPN	
- Yes	245 (75.9%)
- No	70 (21.7%)
- Unknown	8
CVC model	
- BARD—BROVIAC/HICKMAN	210 (65.0%)
- LIFECATH/VYGON	47 (14.6%)
- MEDCOMP	52 (16.1%)
- Other	14 (4.3%)
Insertion side	
- Right	237 (73.4%)
- Left	85 (26.3%)
- Unknown	1
Vein	
- Internal jugular	169 (52.3%)
- External jugular	24 (7.4%)
- Subclavian	50 (15.5%)
- Brachiocephalic	64 (19.8%)
- Other	16 (5.0%)
Dressing setting	
- IRCCS Burlo Garofolo	85.1 (52.71–100.0)
- Satellite hospitals	14.91 (0.0–47.29)
Dressing time interval (% of total dressings)	
- 1–6 days	30.43 (22.2–45.7)
- 7 days	50.0 (36.6–60.1)
- 8–10 days	16.1 (8.9–24.2)

^1^ categorical variables: frequency and percentage. Continuous variables: median and interquartile range. ^2^ categorical variables: frequency and percentage (*n* = 239). CVC: central venous catheter; HSCT: hematopoietic stem cell transplantation; TPN: total parenteral nutrition; IRCCS: “Scientific Research and Healthcare Institute”.

**Table 2:** Proven CLABSI and distribution of main characteristics.

Characteristic	Not Infection, *n* = 299 ^1^	CLABSI, *n* = 24 ^1^	*p*-Value ^2^	OR (95%CI) ^3^
Patient characteristics				
Age (years)	6.9 (3.1, 12.0)	4.1 (2.7, 10.3)	0.2	0.95 (0.88, 1.04)
Sex			0.4	1.5 (0.6, 3.72)
0—Female	114 (38%)	7 (29%)		
1—Male	185 (62%)	17 (71%)		
Diagnosis			0.12	
1—Acute lymphoblastic leukemia	139 (46%)	6 (25%)	0.042	1
2—Acute myeloid leukemia	20 (6.7%)	2 (8.3%)	0.7	2.32 (0.44, 12.28)
3—Myelodysplastic syndromes	22 (7.4%)	4 (17%)	0.11	4.21 (1.1, 16.13)
4—Non-malignant hematological diseases	22 (7.4%)	4 (17%)	0.11	4.21 (1.1, 16.13)
5—Solid Tumors	92 (31%)	8 (33%)	0.8	2.01 (0.68, 6)
6—Unknown	4 (1.3%)	0 (0%)	>0.9	0 (0, Inf)
HSCT			0.4	1.43 (0.62, 3.31)
0—No	188 (63%)	13 (54%)		
1—Yes	111 (37%)	11 (46%)		
CVC characteristics				
CVC Model			0.3	
1—BARD—BROVIAC/HICKMAN	198 (66%)	12 (50%)	0.11	1
2—LIFECATH/VYGON	43 (14%)	4 (17%)	0.8	1.53 (0.47, 4.99)
3—MEDCOMP	46 (15%)	6 (25%)	0.2	2.15 (0.77, 6.04)
4—COOK	1 (0.3%)	0 (0%)	>0.9	0 (0, Inf)
5—Unknown	11 (3.7%)	2 (8.3%)	0.3	3 (0.6, 15.09)
CVC type			>0.9	
1—CICC	277 (93%)	23 (96%)	>0.9	1
2—PICC	13 (4.3%)	1 (4.2%)	>0.9	0.93 (0.12, 7.4)
3—FICC	1 (0.3%)	0 (0%)	>0.9	0 (0, Inf)
4—PORT	8 (2.7%)	0 (0%)	>0.9	0 (0, Inf)
CVC life and use				
CVC duration (days)	237 (111, 325)	163 (58, 272)	0.1	1.00 (0.995, 1.01)
TPN			0.2	0.48 (0.14, 1.65)
0—No	224 (77%)	21 (88%)		
1—Yes	67 (23%)	3 (13%)		
Unknown	8	0		
At least one occlusion			0.9	0.95 (0.41, 2.18)
0—No	133 (44%)	11 (46%)		
1—Yes	166 (56%)	13 (54%)		

^1^ categorical variables: *n* (%); continuous variables: median (IQR). ^2^ Fisher’s exact test; Wilcoxon rank sum test; Pearson’s Chi-squared test. ^3^ univariate logistic regression. CLABSI: central line-associated bloodstream infection; HSCT: hematopoietic stem cell transplantation; TPN: total parenteral nutrition; CICC: Centrally Inserted Central Catheter; PICC: Peripherally Inserted Central Catheter; FICC: Femoral-Inserted Central Catheter; IRCCS: “Scientific Research and Healthcare Institute”.

**Table 3:** Incidence of infection in groups with different dressing frequency.

Group	N. Subjects	CLABSI	Infection Rate per 1000 CVC Days	(95% CI)
Less frequent dressing	111	8	0.31	(0.09–0.53)
Standard dressing	212	16	0.32	(0.16–0.48)

## Data Availability

The original contributions presented in the study are included in the article. Further inquiries can be directed to the corresponding author.

## Supplementary Materials

The following supporting information can be downloaded at: https://www.mdpi.com/article/10.3390/nursrep14040197/s1, Figure S1. CLABSI incidence in the subpopulation with lower dressing frequency. ^1^ For each threshold value on the *x*-axis, we included the subset of patients whose percentage of dressings falling within the 8–10 days range exceeded the threshold. Subsequently, we calculated the incidence of infection for this subset. Incomplete data were removed from the analysis; Table S1: Comparison of variables between CLABSI and non-infection and CVC removed because of infection and other causes.

## Public Involvement Statement

No public involvement in any aspect of this research.

## Guidelines and Standards Statement

This manuscript was drafted against the STROBE guidelines for a cross-sectional study.
