# Supplementary material for: Nurse-Driven Interventions Reduce Central Line-Associated Bloodstream Infection Close to Zero in One Pediatric Oncologic Facility: A Single-Center Retrospective Observational Study
Source: Nurs Rep. 2024 Sep 26;14(4):2668–79. doi: 10.3390/nursrep14040197 (PMC11503445; doi:10.3390/nursrep14040197)
Supplement: Supplementary file 1 [file nursrep-14-00197-s001.zip › nursrep-3154189-supplementary.pdf]

Supplementary Materials

Table S1. Comparison of variables between CLABSI and non-infection and CVC removed because of infection and other causes.

Characteristics	No infection, n = 272[1]	Possible CLABSI, n = 51[1]	p-value[2]	CVC removal for non-infection reasons, n = 292[1]	CVC removal for infection reasons, n = 31[1]	p-value[2]
**Age (years)**	7.1 (3.1, 12.1)	4.3 (2.3, 10.3)	0.085	6.7 (3.0, 11.9)	6.0 (2.9, 11.5)	0.7
**Sex**			0.7			0.8
0 - Female	103 (38%)	18 (35%)		110 (38%)	11 (35%)	
1 - Male	169 (62%)	33 (65%)		182 (62%)	20 (65%)	
**Diagnosis**			0.6			0.12
1 – Acute lymphoblastic leukemia	126 (46%)	19 (37%)	0.2	137 (47%)	8 (26%)	0.025
2 – Acute myeloid leukemia	18 (6.6%)	4 (7.8%)	0.8	19 (6.5%)	3 (9.7%)	0.5
3 - Myelodysplastic syndromes	20 (7.4%)	6 (12%)	0.3	22 (7.5%)	4 (13%)	0.3
4 - Non-malignant hematological diseases	20 (7.4%)	6 (12%)	0.3	21 (7.2%)	5 (16%)	0.089
5 - Solid tumors	84 (31%)	16 (31%)	>0.9	89 (30%)	11 (35%)	0.6
6 - Unknown	4 (1.5%)	0 (0%)	>0.9	4 (1.4%)	0 (0%)	>0.9
**CVC Model**			0.5			0.7
1 - BARD - BROVIAC/HICKMAN	178 (65%)	32 (63%)	0.7	190 (65%)	20 (65%)	>0.9
2 - LIFECATH/VYGON	42 (15%)	5 (9.8%)	0.3	44 (15%)	3 (9.7%)	0.6
3 - MEDCOMP	41 (15%)	11 (22%)	0.2	46 (16%)	6 (19%)	0.6
4 - COOK	1 (0.4%)	0 (0%)	>0.9	1 (0.3%)	0 (0%)	>0.9
5 - Unknown	10 (3.7%)	3 (5.9%)	0.4	11 (3.8%)	2 (6.5%)	0.4
**CVC type**			>0.9			0.9
1 - CICC	251 (92%)	49 (96%)	0.6	271 (93%)	29 (94%)	>0.9
2 - PICC	13 (4.8%)	1 (2.0%)	0.7	13 (4.5%)	1 (3.2%)	>0.9
3 - FICC	1 (0.4%)	0 (0%)	>0.9	1 (0.3%)	0 (0%)	>0.9
4 - Port-a-Cath	7 (2.6%)	1 (2.0%)	>0.9	7 (2.4%)	1 (3.2%)	0.6
**Power Injectable CVC**			0.4			0.6
0 - No	222 (82%)	39 (76%)		237 (81%)	24 (77%)	
1 - Yes	50 (18%)	12 (24%)		55 (19%)	7 (23%)	
**Insertion Method**			0.3			0.6
0 - Percutaneous	232 (88%)	42 (82%)		248 (87%)	26 (84%)	
1 - Surgical	33 (12%)	9 (18%)		37 (13%)	5 (16%)	
Unknown	7	0		7	0	
**Eco-assisted insertion**			0.9			0.3
0 - No	69 (27%)	14 (29%)		77 (29%)	6 (19%)	
1 - Yes	183 (73%)	35 (71%)		193 (71%)	25 (81%)	
Unknown	20	2		22	0	
**Insertion Complications**			0.3			0.3
0 - No	253 (93%)	45 (88%)		271 (93%)	27 (87%)	
1 - Yes	19 (7.0%)	6 (12%)		21 (7.2%)	4 (13%)	
**Subcutaneous Anchor Securement System**			0.6			>0.9
0 - No	247 (91%)	45 (88%)		264 (/90%)	28 (90%)	
1 - Yes	25 (9.2%)	6 (12%)		28 (9.6%)	3 (9.7%)	

**Insertion side**			0.6			>0.9
0 - Right	201 (74%)	36 (71%)		214 (74%)	23 (74%)	
1 - Left	70 (26%)	15 (29%)		77 (26%)	8 (26%)	
Unknown	1	0		1	0	
**N. Lumen**			0.7			>0.9
1	146 (54%)	29 (57%)		158 (54%)	17 (55%)	
2	126 (46%)	22 (43%)		134 (46%)	14 (45%)	
**Diameter (French)**	6.60 (6.00, 7.00)	6.60 (5.00, 7.00)	0.3	6.60 (5.75, 7.00)	6.60 (6.00, 7.00)	0.5
**CVC duration (days)**	234 (112, 326)	204 (59, 306)	0.2	238 (112, 326)	140 (45, 274)	0.019[3]
**HSCT**			0.8			0.4
0 - No	170 (63%)	31 (61%)		184 (63%)	17 (55%)	
1 - Yes	102 (38%)	20 (39%)		108 (37%)	14 (45%)	
**TPN**			0.8			0.2
0 - No	207 (78%)	38 (79%)		218 (77%)	27 (87%)	
1 - Yes	60 (22%)	10 (21%)		66 (23%)	4 (13%)	
Unknown	5	3		8	0	
**Occlusions,** number	1.00 (0.00, 3.00)	1.00 (0.00, 2.00)	0.3	1.00 (0.00, 3.00)	0.00 (0.00, 2.00)	0.3
**Complete Occlusions,** number	0.00 (0.00, 1.00)	0.00 (0.00, 1.00)	0.3	0.00 (0.00, 1.00)	0.00 (0.00, 1.00)	0.4

[1] **number (%); Median (IQR)**

[2] **Pearson's Chi-squared test; Wilcoxon rank sum test; Fisher's exact test**

[3] **The removal of CVC may be considered the cause of reduced CVC duration**

CLABSI: Central-Line Associated Bloodstream Infection; HSCT: Hematopoietic Stem Cell Transplantation; TPN: Total Parenteral Nutrition; CICC: Centrally Inserted Central Catheter; PICC: Peripherally Inserted Central Catheter; FICC: Femoral-Inserted Central Catheter; IRCCS: "Scientific Research and Healthcare Institute";

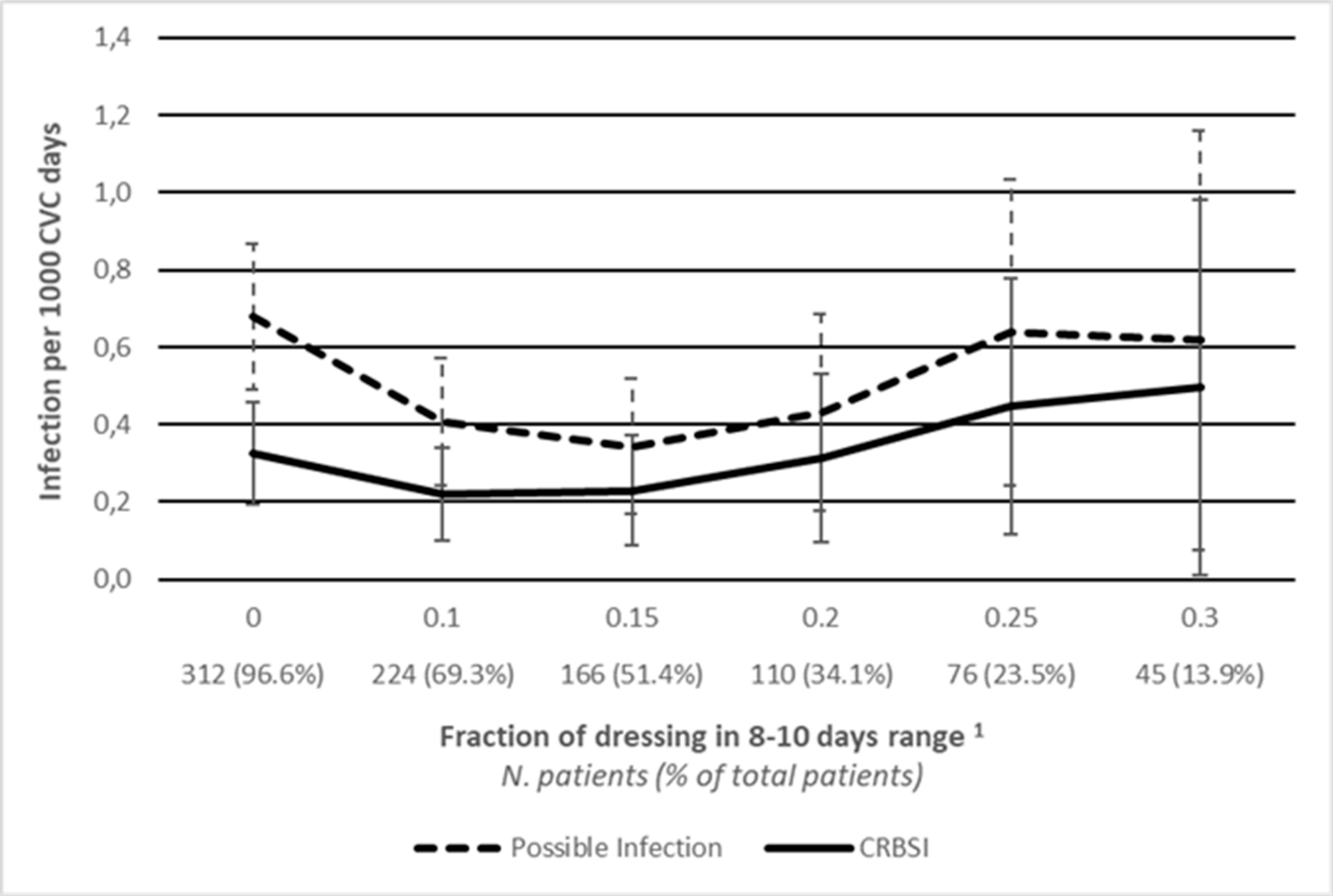

Figure S1. CLABSI incidence in the subpopulation with lower dressing frequency. [1] For each threshold value on the *x*-axis, we included the subset of patients whose percentage of dressings falling within the 8-10 days range exceeded the threshold. Subsequently, we calculated the incidence of infection for this subset. Incomplete data were removed from the analysis.
